# Supplementary material for: A Critical View of the Application of the APEX Software (Aqueous Photochemistry of Environmentally-Occurring Xenobiotics) to Predict Photoreaction Kinetics in Surface Freshwaters
Source: Molecules. 2019 Dec 18;25(1):9. doi: 10.3390/molecules25010009 (PMC7017383; doi:10.3390/molecules25010009)
Supplement: Supplementary file 1 [file molecules-25-00009-s001.zip › 3.2.4_gcc-4.4.0.zip!/3.2.4_gcc-4.4.0/3.2.4_gcc-4.4.0/doc/octave/html/interpreter/The-_003ccode_003eif_003c_002fcode_003e-Statement.html]

The @code{if} Statement - Untitled


Next: The <code>switch</code> Statement,
Up: Statements


---

### 10.1 The `if` Statement

The `if` statement is Octave's decision-making statement. There
are three basic forms of an `if` statement. In its simplest form,
it looks like this:

```
     if (condition)
       then-body
     endif
```

condition is an expression that controls what the rest of the
statement will do. The then-body is executed only if
condition is true.

The condition in an `if` statement is considered true if its value
is non-zero, and false if its value is zero. If the value of the
conditional expression in an `if` statement is a vector or a
matrix, it is considered true only if it is non-empty and *all*
of the elements are non-zero.

The second form of an if statement looks like this:

```
     if (condition)
       then-body
     else
       else-body
     endif
```

If condition is true, then-body is executed; otherwise,
else-body is executed.

Here is an example:

```
     if (rem (x, 2) == 0)
       printf ("x is even\n");
     else
       printf ("x is odd\n");
     endif
```

In this example, if the expression `rem (x, 2) == 0` is true (that
is, the value of `x` is divisible by 2), then the first
`printf` statement is evaluated, otherwise the second `printf`
statement is evaluated.

The third and most general form of the `if` statement allows
multiple decisions to be combined in a single statement. It looks like
this:

```
     if (condition)
       then-body
     elseif (condition)
       elseif-body
     else
       else-body
     endif
```

Any number of `elseif` clauses may appear. Each condition is
tested in turn, and if one is found to be true, its corresponding
body is executed. If none of the conditions are true and the
`else` clause is present, its body is executed. Only one
`else` clause may appear, and it must be the last part of the
statement.

In the following example, if the first condition is true (that is, the
value of `x` is divisible by 2), then the first `printf`
statement is executed. If it is false, then the second condition is
tested, and if it is true (that is, the value of `x` is divisible
by 3), then the second `printf` statement is executed. Otherwise,
the third `printf` statement is performed.

```
     if (rem (x, 2) == 0)
       printf ("x is even\n");
     elseif (rem (x, 3) == 0)
       printf ("x is odd and divisible by 3\n");
     else
       printf ("x is odd\n");
     endif
```

Note that the `elseif` keyword must not be spelled `else if`,
as is allowed in Fortran. If it is, the space between the `else`
and `if` will tell Octave to treat this as a new `if`
statement within another `if` statement's `else` clause. For
example, if you write

```
     if (c1)
       body-1
     else if (c2)
       body-2
     endif
```

Octave will expect additional input to complete the first `if`
statement. If you are using Octave interactively, it will continue to
prompt you for additional input. If Octave is reading this input from a
file, it may complain about missing or mismatched `end` statements,
or, if you have not used the more specific `end` statements
(`endif`, `endfor`, etc.), it may simply produce incorrect
results, without producing any warning messages.

It is much easier to see the error if we rewrite the statements above
like this,

```
     if (c1)
       body-1
     else
       if (c2)
         body-2
       endif
```

using the indentation to show how Octave groups the statements.
See Functions and Scripts.
